# Comment to: Lessons from the pandemic and the value of a structured system of ultrasonographic findings in the diagnosis of COVID-19 pulmonary manifestations

**DOI:** 10.31744/einstein_journal/2024CE1251

**Published:** 2024-10-17

**Authors:** Hinpetch Daungsupawong, Viroj Wiwanitkit

**Affiliations:** 1 Private Academic Consultant Phonhong Lao People's Democratic Republic Private Academic Consultant, Phonhong, Lao People's Democratic Republic.; 2 Chandigarh University University Centre for Research & Development Department of Pharmaceutical Sciences Mohali Punjab India University Centre for Research & Development Department of Pharmaceutical Sciences, Chandigarh University, Mohali, Punjab, India.

Dear Editor,

We aimed to address a paper on the systematic system of ultrasonographic findings for the diagnosis of COVID-19 pulmonary symptoms.^(1)^ The paper described the development of a scoring system to identify lung involvement based on ultrasonography results. Most of the 69 tests were conducted on girls aged 1–96 years. The findings revealed a link between higher COVID-RADS scores and ground-glass opacities on CT, with the majority falling into category 1 and 2. Half of the patients in the upper group required oxygen supplementation. Weaknesses include a small sample size and retrospective approach, which may affect applicability and dependability. However, larger prospective trials are required to validate these findings. Future research should concentrate on observer diversity, the impact on patient outcomes, and the longitudinal assessment of therapeutic interventions using a scoring system.^(2,3)^ These avenues may improve the clinical treatment and further understanding of the evolution of lung involvement in COVID-19.
